# Characterization of Livestock-Associated Methicillin-Resistant *Staphylococcus aureus* Isolates Obtained From Commercial Rabbitries Located in the Iberian Peninsula

**DOI:** 10.3389/fmicb.2018.01812

**Published:** 2018-08-14

**Authors:** Elena Moreno-Grúa, Sara Pérez-Fuentes, Asunción Muñoz-Silvestre, David Viana, Ana B. Fernández-Ros, Celia Sanz-Tejero, Juan M. Corpa, Laura Selva

**Affiliations:** ^1^Biomedical Research Institute (PASAPTA-Pathology Group), Facultad de Veterinaria, Universidad CEU Cardenal Herrera, CEU Universities, Valencia, Spain; ^2^EXOPOL Autovacunas y Diagnóstico, Zaragoza, Spain

**Keywords:** *Staphylococcus aureus*, MRSA, LA-MRSA, rabbit, lesions

## Abstract

Infections caused by methicillin-resistant *Staphylococcus aureus* (MRSA) have been a growing problem in human medicine since the 1960s, and more recently in veterinary medicine with the appearance of livestock-associated MRSA (LA-MRSA). Nevertheless, information about the presence of MRSA in rabbits is quite scarce since only one LA-MRSA identification has been previously reported. The present study aimed to determine genotypic characterization by verifying the presence of resistance determinants, virulence, and toxin genes of different *S. aureus* strains that cause lesions in rabbits, and their phenotypic traits based on the antimicrobial susceptibility profile. The analysis of 240 *S. aureus* isolates obtained from different lesion types collected from 89 Spanish and Portuguese rabbit commercial farms in the last 4 years (2014–2017) was performed. The methicillin-resistant gene *mec*A was found in 11.25% of the studied isolates (27 of 240) from 19 farms (13 Spanish and 6 Portuguese). Staphylococcal cassette chromosome *mec* (SCC*mec*) typing predominantly revealed type III (*n* = 15). Additionally, three MRSA isolates carrying the *mec*C gen were detected in samples from three different farms (two Spanish and one Portuguese). None of the 30 MRSA isolates was *PVL*-positive or *tst*-positive. After the multilocus sequence typing (MLST) procedure, 16 belonged to ST2855, 6 to ST146, 6 to ST398, and 2 ST4774. No ST121 isolate was *mec*-positive. ST398 and ST4774 isolates lacked the immune-evasion-cluster (IEC) genes. ST2855 strains were associated with the presence only of the *sak* gene, and ST146 isolates were ascribed to IEC type E. Therefore, this is the first description of LA-MRSA from rabbits belonging to ST2855. Interestingly, one ST2855 and two ST4774 isolates were *mec*C-positive, which could act as a *mec*C-MRSA reservoir. More studies are needed to further characterize these isolates and their relationship with humans and other animal species.

## Introduction

*Staphylococcus aureus* multi-resistant to antibiotics is a leading cause of bacterial infections in hospitals and communities. Specifically, methicillin-resistant *S. aureus* (MRSA) has spread worldwide in the second half of the 20th century and is now considered endemic in healthcare facilities in all industrialized countries (Kobayashi et al., 2015). In 2005 livestock-associated MRSA (LA-MRSA) emerged in pigs (Armand-Lefevre et al., 2005), where it plays an important role a reservoir of infection to humans (Frana et al., 2013). It was later reported in different farm animals, including horses, cattle or poultry worldwide (Graveland et al., 2011; Aires-de-Sousa, 2016). *S. aureus* frequently infects commercial rabbits (Corpa et al., 2009), but there is only one reported case in which LA-MRSA was identified in this animal species, and was also isolated from farmers and their relatives (Agnoletti et al., 2014).

The results of population genetics studies have shown that most *S. aureus* strains are host-specific, which indicates low frequency of cross-species transmission (Fitzgerald, 2012). However, more recent studies that employed the multilocus sequence typing (MLST) have identified several sequence types (ST) that are associated with multiple host species. This finding implies either zoonotic transmission or a recent common ancestor (Spoor et al., 2013).

Several *S. aureus* MLST lineages have been associated with animals, including clonal complexes (CC): CC1 (livestock), CC5 (avian), CC130 (multi-host), CC133 (ruminants), CC151 (ruminants), CC398 (livestock), and CC425 (ruminants and wild mammals) (Harrison et al., 2017). *S. aureus* CC121 has a multi-host tropism (Viana et al., 2015a), including humans, where it is considered a globally disseminated hypervirulent clone, although 90% of ST121 strains are methicillin-sensitive (Rao et al., 2015). In commercial rabbits, ST121 is by far the most frequently isolated clone, with prevalences close to, and even higher than, 90% (Viana et al., 2011; Guerrero et al., 2015). This causes important economic loss associated with several lesions, such as mastitis, multisystemic abscessation and pododermatitis (Corpa et al., 2009). However, no information in rabbits about the susceptibility of ST121 strains to methicillin is available.

Due to the increasing presence of LA-MRSA strains in different animal species and the scarcity of available information on commercial rabbits, the aims of this study were to: (i) evaluate the presence of MRSA in a *S. aureus* collection obtained from staphylococcal lesions on different Spanish and Portuguese rabbit farms; (ii) compare the isolated strains using MLST, SCC*mec*, and *agr* typing; (iii) determine antibiotic resistance profile and virulence factors.

## Materials and Methods

### Isolation and Characterization of *S. aureus* Isolates

Two hundred and forty *S. aureus* isolates from rabbit clinical samples obtained on 89 rabbitries located in Spain (*n* = 82) and Portugal (*n* = 7), were studied in our laboratories in the last 4 years (2014–2017). These selected samples came from rabbits with different lesion types, including mastitis (*n* = 86), subcutaneous abscesses (*n* = 33), pododermatitis (*n* = 31), dermatitis (*n* = 21), otitis (*n* = 13), metritis (*n* = 12), conjunctivitis (*n* = 11), pneumonia (*n* = 9), rhinitis (*n* = 3), hepatitis (*n* = 3), peritonitis (*n* = 2), pericarditis (*n* = 1), and osteomyelitis (*n* = 1). Fourteen *S. aureus* isolates from nasal carriers were also analyzed.

Samples were inoculated on blood-agar (BioMérieux, Marcy l’Etoile, France) and incubated aerobically at 37°C for 24–48 h. *S. aureus* strains were identified on the basis of morphological growth characteristics and hemolytic properties (Devriese et al., 1996). To perform PCR, genomic DNA was extracted from each isolate with a Genelute Bacterial Genomic DNA kit (Sigma), according to the manufacturer’s protocol, except for bacterial cells, which were lysed by lysostaphin (12.5 μg/ml, Sigma) at 37°C for 1 h before DNA purification. Isolates were genotyped by MLST (Enright et al., 2000).

All the strains were checked for the presence of the *mec*A/*mec*C genes by PCR, as previously described (Geha et al., 1994; Khairalla et al., 2017). Furthermore, SCC *mec* cassette element classification was carried out for all the *mec*A-positive isolates, as described elsewhere (Zhang et al., 2005; Milheirico et al., 2007).

### Detection *chp*, *sak*, *sea*, *sep*, *scn*, *tst,* and *PVL*-Encoding Genes and *agr* Typing

The MRSA isolates were subjected to a PCR assay to detect the *lukF/S-PV* genes that encode the PVL toxin and the *tst* gene that encodes the TSST-1 toxin and *agr* typing, as previously described (Viana et al., 2015b). These strains were also checked for the presence of the immune-evasion cluster (IEC) genes (*sea*, *sep*, *sak*, *chp*, and *scn*) by PCR, as formerly described (van Wamel et al., 2006).

### Antibiotic Susceptibility Testing

The antibiotic susceptibility of the MRSA isolates was determined by the disk diffusion method on Mueller-Hinton agar (MHA, CONDA, Spain), according to the recommendations of the Clinical and Laboratory Standards Institute (CLSI). The disk diffusion assay was done with 14 antibiotics: bacitracin (10 U), enrofloxacin (5 μg) (OXOID), streptomycin (10 μg), spiramycin (100 μg), sulfadiazine (25 μg), chloramphenicol (30 μg) (BD), doxycycline (30 μg), erythromycin (15 μg), gentamicin (10 μg), neomycin (30 μg), penicillin (10 U), tetracycline (30 μg), cefoxitin (30 μg), and trimethoprim/sulfamethoxazole (1.25 μg/23.75 μg, respectively) (BIO-RAD). Minimum inhibitory concentrations (MIC) for cefoxitin and vancomycin was determined by using MIC Test Strip (Liofilchem) on inoculated Mueller Hinton agar plates and the results were interpreted according EUCAST breakpoints. *S. aureus* strain ATCC 25923 and *Enterococcus faecalis* strain ATCC 29212 were used as controls in the susceptibility test.

### Statistical Analysis

Categorical data were compared using Fisher exact test. All reported *p*-values are two-tailed and analyses were performed using GraphPad software.^1^ Variables with *p* < 0.01 were considered to be statistically significant.

## Results

### Identification of MRSA and Lesions

Of all the analyzed *S. aureus* isolates, 12.5% (30/240) were identified as methicillin-resistant. Of the 30 MRSA isolates, *mec*A was detected in 27 isolates. In 3 isolates *mec*C were amplified. The 30 isolates were cefoxitin-resistant, with inhibition zone diameters <22 mm (range 0–20 mm) using disk diffusion method and MIC values >4 μg/ml (range 8–256 μg/ml).

When considering lesion type, MRSA was most frequently present in hepatitis samples (100%, 3/3), followed by metritis (58.3%, 7/12), pneumonia (44.4%, 4/9), rhinitis (33.3%, 1/3), otitis (23.1%, 3/13), conjunctivitis (18.2%, 2/11), mastitis (9.3%, 8/86), dermatitis (4.8%, 1/21), and pododermatitis (3.2%, 1/31). Hepatitis and metritis were strongly associated with MRSA (*p* < 0.0001) (see **Table 1**). When only the commonest lesions (mastitis, abscesses, and pododermatitis) were taken into account, MRSA was identified in 6% (9/150) of the cases. None of the 14 isolates from nasal carriers were MRSA.

**Table 1:** Number of MRSA and MSSA isolates identified from different lesions.

Lesion	No. isolates	MRSA	MSSA	*p-*value
Mastitis	86	8	78	0.3127
Abscess	33	–	33	0.0192
Pododermatitis	31	1	30	0.1422
Dermatitis	21	1	20	0.4867
Otitis	13	3	10	0.2120
Metritis	12	7	5	**0.0001**
Conjunctivitis	11	2	9	0.6328
Pneumonia	9	4	5	0.0162
Rhinitis	3	1	2	0.3313
Hepatitis	3	3	0	**0.0018**
Peritonitis	2	–	2	1
Pericarditis	1	–	1	1
Osteomyelitis	1	–	1	1
Nasal carrier	14	–	14	0.2270
**Total**	**240**	**30**	**210**	


Bolded values indicate variables with p < 0.01 were considered to be statistically significant.

### Characterization of *S. aureus* Isolates

The MLST typing analysis revealed 13 different STs. The MLST type, including the largest number of isolates, was ST121 (*n* = 73). This was immediately followed by ST3764 (*n* = 57), ST96 (*n* = 36), ST2855 (*n* = 32), and ST1 (*n* = 13). Other less frequent STs were ST398 (*n* = 11), ST146 (*n* = 6), ST5 (*n* = 4), ST3761 (*n* = 3), ST4774 (*n* = 2), a novel *tpi* single locus variant of ST130, ST4473 (*n* = 1), a novel *arc* single locus variant of ST4470, ST407 (*n* = 1), and ST3759 (*n* = 1).

The 30 MRSA isolates from 22 farms (15 Spanish and 7 Portuguese) belonged to ST2855 (53.3%, 16/30), ST146 (20%, 6/30), ST398 (20%, 6/30), and ST4774 (6.6%, 2/30). For SCC*mec* typing, the multiplex PCR assay identified 15 of the 27 *mec*A MRSA isolates with SCC*mec* type III, 6 with SCC*mec* type IV, and 6 with SCC*mec* type V. The 3 *mec*C MRSA isolates belonged to a ST4774 and one ST2855.

All the MRSA strains belonging to the same lineage displayed an identical accessory gene regulator (*agr*) type and SCC*mec*-type: ST2855 harbored *agr*III and SCC*mec*-type III; ST146 displayed *agr*II and SCC*mec*-type IV; ST398 contained *agr*I and SCC*mec*-type V; ST4774 harbored *agr*III (**Table 2**). Methicillin-susceptible *S. aureus* (MSSA) strains with *agr* type I belonged to lineage ST398 and ST407; ST5 and ST3759 harbored *agr*II; ST1, ST96, ST2855, and ST4473 displayed *agr*III; ST121, ST3764, and ST3761 contained *agr*IV.

**Table 2:** Relationship among the type of lesions and genotypic (MLST, SCC*mec*, *agr* and IEC types) and phenotypic (antibiotic profile) characteristics in the MRSA isolates.

Isolate ID	Lesion		Genotypic characteristics			MIC (μg/ml)		Phenotypic resistance profile^∗^
		ST (MLST)	SCC*mec* type	*agr* types	IEC types	FOX	VAN	
Sp-794	Otitis	146	IV	II	E	32	0.75	ENO-N-TET
Sp-795	Otitis	146	IV	II	E	24	1.5	ENO-N-TET
Sp-986	Dermatitis	146	IV	II	E	48	1.5	N-TET
Sp-992	Otitis	146	IV	II	E	48	1.5	ENO-S-N-TET
Sp-1004	Pneumonia	146	IV	II	E	48	1.5	ENO-N-TET
Sp-1005	Pneumonia	146	IV	II	E	48	2	ENO-S-N
Sp-1006	Conjunctivitis	398	V	I	–	16	0.75	CN-TET
Sp-1007	Rhinitis	398	V	I	–	12	1	ENO-E-SPC-TET
Sp-1008	Mastitis	398	V	I	–	16	0.75	DO-ENO-E-SPC-TET
Sp-1018	Mastitis	398	V	I	–	12	0.75	ENO-E-SPC-CN-TET
Sp-1019	Conjunctivitis	398	V	I	–	12	0.75	ENO-E-SPC-CN-TET
Sp-1032	Mastitis	398	V	I	–	12	0.75	ENO-S-TET
P-988	Metritis	2855	III	III	–	16	1.5	S-TET
P-989	Mastitis	2855	III	III	–	32	1.5	ENO-E-SPC-S
Sp-990	Pneumonia	2855	III	III	–	32	1.5	ENO-E-SPC-S-TET
P-991	Metritis	2855	III	III	–	32	1.5	ENO-E-SPC-S-TET
Sp-993	Mastitis	2855	III	III	–	16	1.5	S
P-994	Pododermatitis	2855	III	III	–	32	1	C-ENO-E-SPC-TET
P-995	Hepatitis	2855	III	III	–	48	1.5	ENO-S-N
P-996	Mastitis	2855	III	III	–	32	2	ENO-E-SPC-S-TET
P-997	Mastitis	2855	III	III	–	48	2	DO-ENO-E-SPC-S-TET
P-998	Metritis	2855	III	III	–	32	1	DO-E-SPC-TET
Sp-1000	Metritis	2855	III	III	–	48	2	ENO-E-SPC-S-TET
Sp-1001	Hepatitis	2855	III	III	–	256	1.5	ENO-E-S-TET
Sp-1002	Metritis	2855	III	III	–	32	1.5	ENO-E-SPC-S-TET
P-1003	Hepatitis	2855	III	III	–	64	2	DO-E-SPC-S-TET
P-1009	Pneumonia	2855	III	III	–	48	2	CN-TET
P-985	Metritis	2855	*mec*C	III	–	12	1.5	C-ENO -E-SPC-S
Sp-987	Metritis	4774	*mec*C	III	–	8	1	ENO-E-SPC-S-TET
Sp-999	Mastitis	4774	*mec*C	III	–	12	1.5	ENO-E-SPC-TET


^∗^All MRSA strains were resistant to cefoxitin and penicillin and susceptible sulfonamides (sulfadiazine and trimethoprim/sulfamethoxazole) and bacitracin. C, chloramphenicol; CN, gentamicin; DO, doxycycline; E, erythromycin; ENO, enrofloxacin; FOX, cefoxitin; MIC, Minimum inhibitory concentrations; N, neomycin; P, Portugal; S, streptomycin; Sp, Spain; SPC, spiramycin; TET, tetracycline; VAN, vancomycin.

### Antibiotic Resistance Profile

The full resistance rates among the 30 MRSA isolates tested in the present study were as follows: 100% (*n* = 30) for cefoxitin and penicillin, 83.3% (*n* = 25) for tetracycline, 76.6% (*n* = 23) for enrofloxacin, 60% (*n* = 18) for erythromycin, 56.6% (*n* = 17) for streptomycin and spiramycin, 23.3% (*n* = 7) for neomycin, 13.3% (*n* = 4) for doxycycline and gentamicin and 6.6% (*n* = 2) for chloramphenicol. Conversely, all the tested isolates were susceptible to sulfonamides (sulfadiazine and trimethoprim/sulfamethoxazole) and bacitracin.

The majority of the tested MRSA isolates (*n* = 29, 96.6%) were multidrug-resistant (resistant to three antimicrobial classes or more). A comparison of occurrence of antimicrobial resistance among the investigated MRSA isolates in relation to different STs is presented in **Table 2**. The ST2855 strains showed resistance to a larger number of antibiotic groups than other MRSA strains; specifically to tetracycline, macrolides (erythromycin and spiramycin) and enrofloxacin. The highest percentage of tetracyclines (100%) resistance was recorded among the ST398 strains. ST146 isolates showed resistance to a smaller number of antibiotics, of which neomycin (100%), enrofloxacin (83.3%), and tetracycline (83.3%) stood out. The two ST4774 isolates showed resistance to enrofloxacin, macrolides, and tetracycline (100%) and in one case also to streptomycin. For the remaining antimicrobial groups, no differences in resistance were observed among the STs. MIC Test Strip for cefoxitin detect a strain ST2855 with high level resistance (MIC, 256 μg/ml). None of the MRSA isolates showed resistance to vancomycin, with MICs ranged from 0.5 to 2 μg/ml.

### Detection of IEC Cluster (*scn*, *chp*, *sak*, and *sea* or *sep*), *tst*, and PVL Genes Among MRSA Isolates

The PCR detection of the IEC genes, *tst* and PVL-encoding genes was carried out for the MRSA isolates. None of the MRSA isolates were positive for *sea* or *sep*, *chp*, *tst,* and PVL genes. The *sea*/*sep* gene along with the *sak* (73.3%), *chp* (0%), and *scn* (20%) genes, modulators of different parts of the innate immune system, forming an immune evasion cluster (IEC) (van Wamel et al., 2006). Depending on the presence or absence of these genes and their different combinations, *S. aureus* isolates were classified into 7 different IEC types according to patters previously described. The *scn* gene is mandatory for the consideration of the IEC types (Benito et al., 2016). All ST146 isolates contained IEC type E (comprised of *sak* and *scn* genes). However, isolates belonging to ST2855 were associated with the presence only of the *sak* gene, while the strains belonging to ST398 and ST4774 did not contain IEC genes.

## Discussion

In the present study, 240 *S. aureus* isolates obtained from rabbits suffering different lesions, located on 89 farms of Spain and Portugal, were analyzed between 2014 and 2017. Isolates were obtained mainly from mastitis (*n* = 86), abscesses (*n* = 33) and pododermatitis (*n* = 31), which were the most frequently observed lesions associated with *S. aureus* infections in commercial rabbits (Segura et al., 2007; Viana et al., 2007).

Screening for methicillin-resistant isolates allowed the identification of 30 strains as MRSA. The lesion types from which they were isolated differed compared with those upper previously indicated as more frequent (mastitis, abscesses, and pododermatitis). MRSA was detected in 100% (3 out of 3) of hepatitis cases, metritis (58.3%, 7/12), pneumonia (44.4%, 4/9), rhinitis (33.3%, 1/3), otitis (23.1%, 3/13), conjunctivitis (18.2%, 2/11), mastitis (9.3%, 8/86), dermatitis (4.8%, 1/21), and pododermatitis (3.2%, 1/31). Therefore, while the percentage of the most frequent lesions (mastitis, abscesses, and pododermatitis) caused by *S. aureus* was 62.5% of all lesions (150/240), MRSA was involved only in 30% (9/30) of these same lesions. They were not isolated from abscesses and there was only one pododermatitis case. Hepatitis and metritis were found to be strongly associated with MRSA (hepatitis *p* < 0.0018; metritis *p* < 0.0001). This unusual lesion pattern occasioned with MRSA strains *versus* habitual *S. aureus* infections could indicate a different pathogenesis of MRSA infections in rabbits.

In order to understand the pathogenesis of staphylococcal infections, the correct identification of the involved strain is vital. Thus the development of high discriminatory typing techniques, such as MLST, is very important (Enright et al., 2000). In the present study, the most frequently detected MLST type was ST121 (73/240). It has been reported that the majority of chronic staphylococcal infections in rabbits are caused by high virulence strains that belong mainly to the ST121 lineage (Vancraeynest et al., 2006; Viana et al., 2011; Guerrero et al., 2015). The ST121 lineage was also the predominant one in this study, followed immediately by ST3764 (57/240). This last lineage, together with strains ST3761, a minority in our study (*n* = 3), belong to clonal complex CC121. *S. aureus* CC121 has a multi-host tropism, which is a common cause of human skin and soft-tissue infections (Viana et al., 2015a). In humans, the ST121 lineage is also considered a hypervirulent clone, although approximately 90% of the ST121 strains were methicillin-sensitive (Rao et al., 2015). The present study agrees with these results since all the ST121 strains from rabbit isolates were methicillin-sensitive.

A further ten different ST types among the tested isolates were identified: ST96 (*n* = 36), ST2855 (*n* = 32), ST1 (*n* = 13), ST398 (*n* = 11), ST146 (*n* = 6), ST5 (*n* = 4), ST4774 (*n* = 2), ST4473 (*n* = 1), ST407 (*n* = 1), and ST3759 (*n* = 1). However, MRSA isolates showed limited genetic diversity (ST2855, ST146, ST398, and ST4774), being the ST2855 the most predominant clone (53.3%; 16/30). It is noteworthy that 50% (16/32) of strains ST2855 were methicillin-sensitive. The only isolate described to date to belong to this lineage is an MSSA isolate of mastitis, described in a rabbit from Italy in 2012.^2^ All the ST146 strains isolated herein were methicillin-resistant. No data exists in the literature about *S. aureus* isolates from rabbits that belong to this lineage. ST146 belongs to clonal complex CC5. This clonal complex has been previously found in *S. aureus* isolates from rabbit carcasses, but none were methicillin-resistant (Merz et al., 2016). *S. aureus* associated with CC5 is commonly detected in humans or animal hosts, including poultry (Krupa et al., 2018). On the other hand, six of the 11 ST398 isolates were MRSA. The only LA-MRSA case to have occurred in rabbits for meat production belongs to this lineage (Agnoletti et al., 2014). Finally, the 3 *mec*C MRSA isolates belonged to ST4774, a novel *tpi* single locus variant of ST130, and one ST2855. Isolates reported to date and carrying *mec*C belonged mainly to lineages common in cattle, namely CC130, CC1943, and CC425, suggesting a zoonotic reservoir (Aires-de-Sousa, 2016). Besides cattle, *mec*C has also been found among other farm animals such isolates ST130 in sheep (Ariza-Miguel et al., 2014; Giacinti et al., 2017) and an isolate ST425 that caused a highly virulent infection in a rabbit (Paterson et al., 2012).

Other typing techniques used to characterize MRSA strains include the identification of the *agr* and staphylococcal cassette chromosome *mec* (SCC*mec*). The *mec*A-positive MRSA strains that belong to the same lineage displayed identical *agr* and *mec* types: ST2855 harbored *agr*III and *mec* type III; ST146 displayed *agr*II and *mec* type IV and ST398 contained *agr*I and *mec* type V. The *mec*C-positive isolates harbored *agr*III. The *agr* locus plays a critical role in MRSA pathogenesis and has been assumed to play a key role in human staphylococcal infections (El-Baz et al., 2017). Other lineages with *agr*III include strains type ST96 or ST1. In rabbits, differences have been detected in the virulence between strains ST121 and ST96 (Guerrero et al., 2015; Viana et al., 2015b), which could be related with differences with this regulator type. Regarding the SCC*mec* type, different combinations of genes in several SCC*mec* types also leads to the strains displaying distinct antibiotic susceptibilities. Although the majority of the tested MRSA isolates were multidrug-resistant, the ST2855 strains showed resistance to a larger number of antibiotic groups than other strains, as well as higher frequency of resistance to macrolides. This multiresistance could justify its more extended presence compared to other MRSA strains. Types II and III SCC*mec* segments have been reported to be longer and to possess multidrug resistances to the strains that carry these elements. These types have frequently been demonstrated in HA-MRSA strains (Taherirad et al., 2016). Similarly to other studies, the ST398 strains identified in this study belonged to V SCC*mec* type (Witte et al., 2007; Bardiau et al., 2013). The highest percentage of tetracyclines (100%) resistance were recorded among the ST398 strains. Resistance to tetracycline is also associated with LA ST398 (Harrison et al., 2017), and acquisition of *tet*K as part of SCC*mec* type Vc by *tet*M-positive LA ST398 has been demonstrated (Larsen et al., 2016). On the other hand, ST146 isolates showed resistance to fewer antibiotics, of which neomycin (100%), enrofloxacin (83.3%) and tetracycline (83.3%) stand out. In Spain, ST146 MRSA has been detected in nasal carriage in Primary Healthcare Center patients. This isolate was typed as ST146-CC5 SCC*mec* IVc, and it presented a multiresistance phenotype, similarly to our ST146 rabbit strains. Strains with similar characteristics are considered hospital-acquired HA-MRSA (Lozano et al., 2015). Finally, *mec*C is part of a novel SCC*mec* assigned type XI (García-Álvarez et al., 2011). Aires-de-Sousa (2016) describes that isolates of this new genetic element are not resistant to antibiotics other than beta-lactams, in contrast to our results where *mec*C strains also showed resistance to enrofloxacin, tetracycline and macrolides.

Finally, all the MRSA strains described in this study were negative for the genes that code toxins PVL and TSST-1. A comparative analysis of the accessory genomes of the ST121 strains showed that the majority of human strains contained mobile genetic elements, which encode the potent toxins involved in human disease pathogenesis, such as PVL and TSST-1. According to our study, no rabbit strains carried *luk*S/F-PV or *tst* genes, which indicates that they are dispensable for the *S. aureus* infection of rabbits (Viana et al., 2015a). On the other hand, the presence of the immune-evasion-cluster (IEC) genes was determined for MRSA isolates to determine whether they may have human or animal origin. The presence of IEC type E in all ST146 isolates points to the possible human origin of this clone. However, ST398 and ST4774 (a single locus variant of ST130) isolates belonging to LA-lineages since were IEC negative. There are numerous reports of the presence of LA-MRSA in livestock caused by CC398 and several MRSA lineages as CC130 (Agnoletti et al., 2014; Paterson et al., 2014; Aires-de-Sousa, 2016; Harrison et al., 2017). However, isolates belonging to ST2855 were associated with the presence only of the *sak* gene. Stegger et al. (2013) described the presence of IEC in a porcine *S. aureus* CC398 isolate within the livestock clade, which supports that reacquisition of IEC enables LA-MRSA CC398 to spread in human populations. More recently Kraushaar et al. (2017) demonstrated that lysogenic conversion of LA-CC398 strains by virulence-associated phages may occur and that new pathotypes may emerge by this mechanism.

Vancomycin is an important antibiotic to treat MRSA isolates, so the emergence of vancomycin-resistant *S. aureus* (VRSA) strains poses a serious global threat to public health. Two mechanisms, including cell wall changes and acquired *van* genes were involved in vancomycin resistance. None of the MRSA isolates showed resistance to vancomycin.

A cefoxitin disk diffusion test is used to predict the presence of *mec*A in *S. aureus* (Cauwelier et al., 2004; Swenson et al., 2005). All MRSA isolates were cefoxitin-resistant, with inhibition zone diameters <22 mm using disk diffusion method. Therefore, this method is a good test for routine detection of all classes of MRSA. On the other hand, isolates harboring *mec*C typically yield negative results for conventional *mec*A PCR, which can translate in detection errors in a subset of MRSA isolates (Paterson et al., 2012). Therefore, detecting *mec*A/*mec*C genes proves to be more reliable test for detecting methicillin resistance among staphylococci.

Methicillin-resistant *S. aureus* prevalence varies according to sampling type and the studied animal species. The European Food Safety Authority [EFSA] (2009) has reported a 26.9% MRSA prevalence, which was detected in the dust of pigs’ production holdings of the European Union. Recent research into small ruminants has indicated an overall estimated prevalence of 0.70% (2/286) in milk samples in herds (Giacinti et al., 2017). In cattle a 4.4% prevalence has been reported in isolates collected from bovine mastitis cases (Bardiau et al., 2013). In the only MRSA case published in rabbits, only in 1 of 40 (2.5%) farms with clinical staphylococcosis has detected MRSA in skin samples (Agnoletti et al., 2014). Although the present study was not designed according to an epidemiological point of view, an unexpectedly large number of positive samples (12.5%; 30 of 240 isolates) from 22 different farms (22 of 89 farms) in Spain (*n* = 15) and Portugal (*n* = 7) was detected. Therefore, it can be stated that the spread of MRSA strains on Spanish and Portuguese rabbitries in the last 4 years is worrisome.

## Conclusion

An unexpected large number of MRSA strains obtained from numerous rabbit farms, isolated from infrequent lesions in *S. aureus* infections, is herein reported. A new lineage of MRSA, ST2855 associated with livestock, has not yet been described. This is the first description of LA-MRSA in rabbits belonging to ST2855 and the first report of *mec*C MRSA in rabbit samples from Iberian Peninsula. More studies are needed to further characterize these isolates and their relationship with humans and other species.

## Ethics Statement

No approval from the Animal Welfare Ethics Committee was required, since all isolates were analyzed as part of microbiological diagnostics in accordance with the Spanish law RD53/2013.

## Author Contributions

JC and LS conceived the presented idea. EM-G, SP-F, and AM-S carried out the experiments. AF-R and CS-T contributed to samples preparations. LS wrote the manuscript with support from DV and JC. All authors discussed the results and contributed to the final manuscript.

## Conflict of Interest Statement

The authors declare that the research was conducted in the absence of any commercial or financial relationships that could be construed as a potential conflict of interest.

## Footnotes

1 https://www.graphpad.com/quickcalcs/catMenu/

2 https://pubmlst.org/saureus/
